# Spectral responses across a dorsal–ventral array of dermal sensilla in the medicinal leech

**DOI:** 10.1007/s00359-021-01508-z

**Published:** 2021-09-03

**Authors:** Thomas K. H. Groves, John A. Jellies

**Affiliations:** Department of Biological Sciences, Western Michigan University, Kalamazoo, MI 49008 USA

**Keywords:** Leech, Ultraviolet, Sensilla, Photoreceptors, Vision

## Abstract

How do animals use visual systems to extract specific features of a visual scene and respond appropriately? The medicinal leech, *Hirudo verbana*, is a predatory, quasi-amphibious annelid with a rich sensorium that is an excellent system in which to study how sensory cues are encoded, and how key features of visual images are mapped into the CNS. The leech visual system is broadly distributed over its entire body, consisting of five pairs of cephalic eyecups and seven segmentally iterated pairs of dermal sensilla in each mid-body segment. Leeches have been shown to respond behaviorally to both green and near ultraviolet light (UV, 365–375 nm). Here, we used electrophysiological techniques to show that spectral responses by dermal sensilla are mapped across the dorsal–ventral axis, such that the ventral sensilla respond strongly to UV light, while dorsal sensilla respond strongly to visible light, broadly tuned around green. These results establish how key features of visual information are initially encoded by spatial mapping of photo-response profiles of primary photoreceptors and provide insight into how these streams of information are presented to the CNS to inform behavioral responses.

## Introduction

Visual systems evolved in response to the ubiquity of electromagnetic radiation across many environments, providing a significant source of sensory information to a wide variety of organisms. We are primarily interested in how the organization and distribution of visual receptors might impact behavior. To understand the adaptive value of visual systems in general and how they evolved, mechanisms of image formation and processing must be considered (Fernald 2000; Arendt 2003; Nilsson 2009). From the valuable insights provided by the work of Hartline (1938), Kuffler (1953), and Hubel and Wiesel (1962), we know that image formation, even in animals with high-resolution image-forming eyes, is based on the mapping of somewhat abstract features that are extracted by neural circuitry. To date, examination of how visual systems encode and compute these visual features continues to excite and challenge researchers. Color vision in light-attenuating environments (for example aquatic, or densely shaded terrestrial), the use of ultraviolet light as a visual cue, spatial vision and motion detection, and the behavioral consequences of vision are important issues in the neurobiology of visual systems (Tovée 1995; Cronin et al. 2014). Defining the mechanisms of feature extraction and processing is aided by appreciating systems with experimentally tractable eyes, neural circuitry, and synaptic integration.

**Fig. 1:**
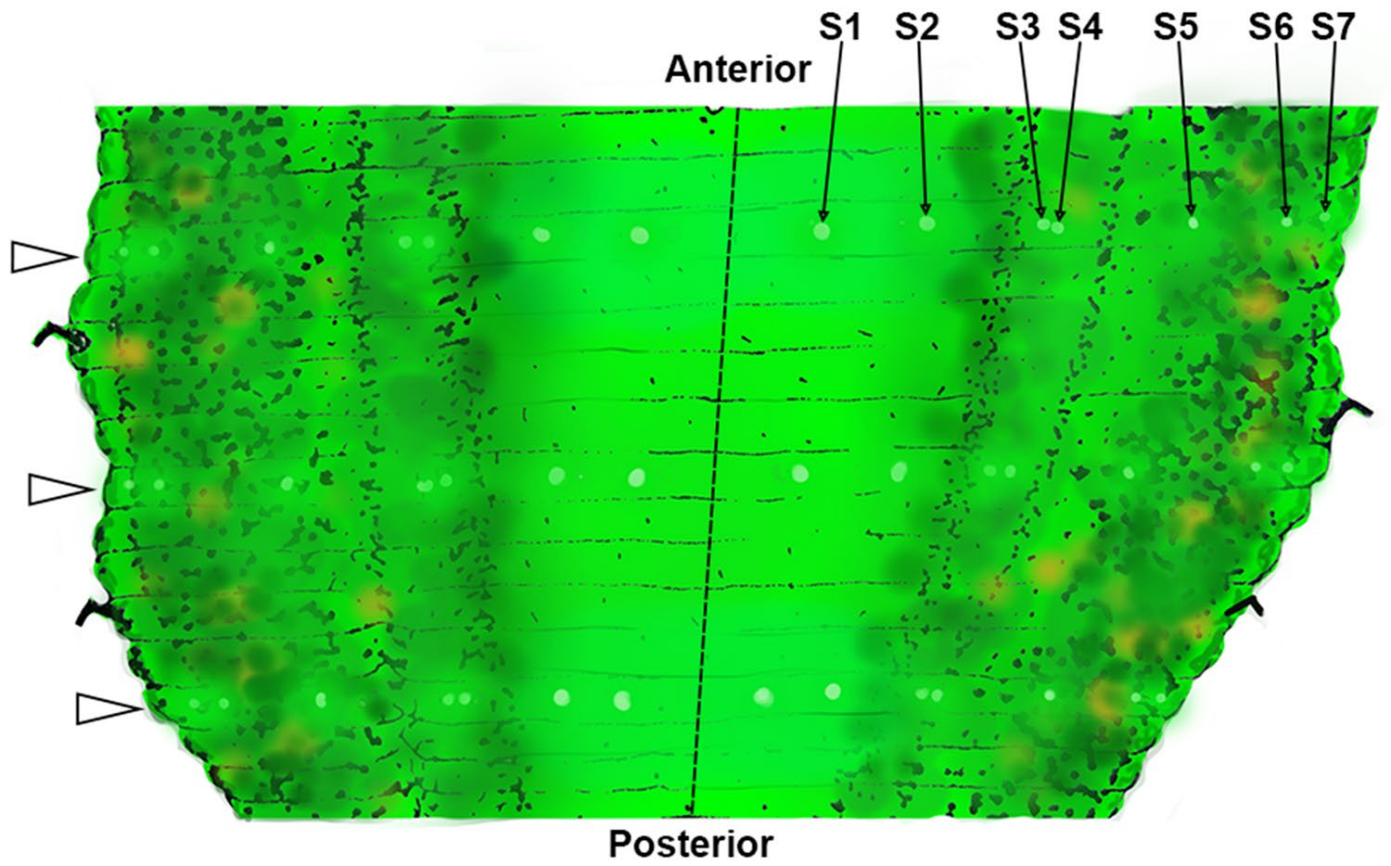
Artistic representation of several mid-body segments as they would appear in the preparations described in this work. The dashed line represents the ventral midline, while the lateral edges of the figure represent the cut dorsal midline that has been pulled apart and pinned during dissection. Open white arrows indicate the central annuli of the three representative mid-body segments, with the black arrows pointing to sensilla 1–7 on the left side of the animal within the anterior-most central annulus

**Fig. 2:**
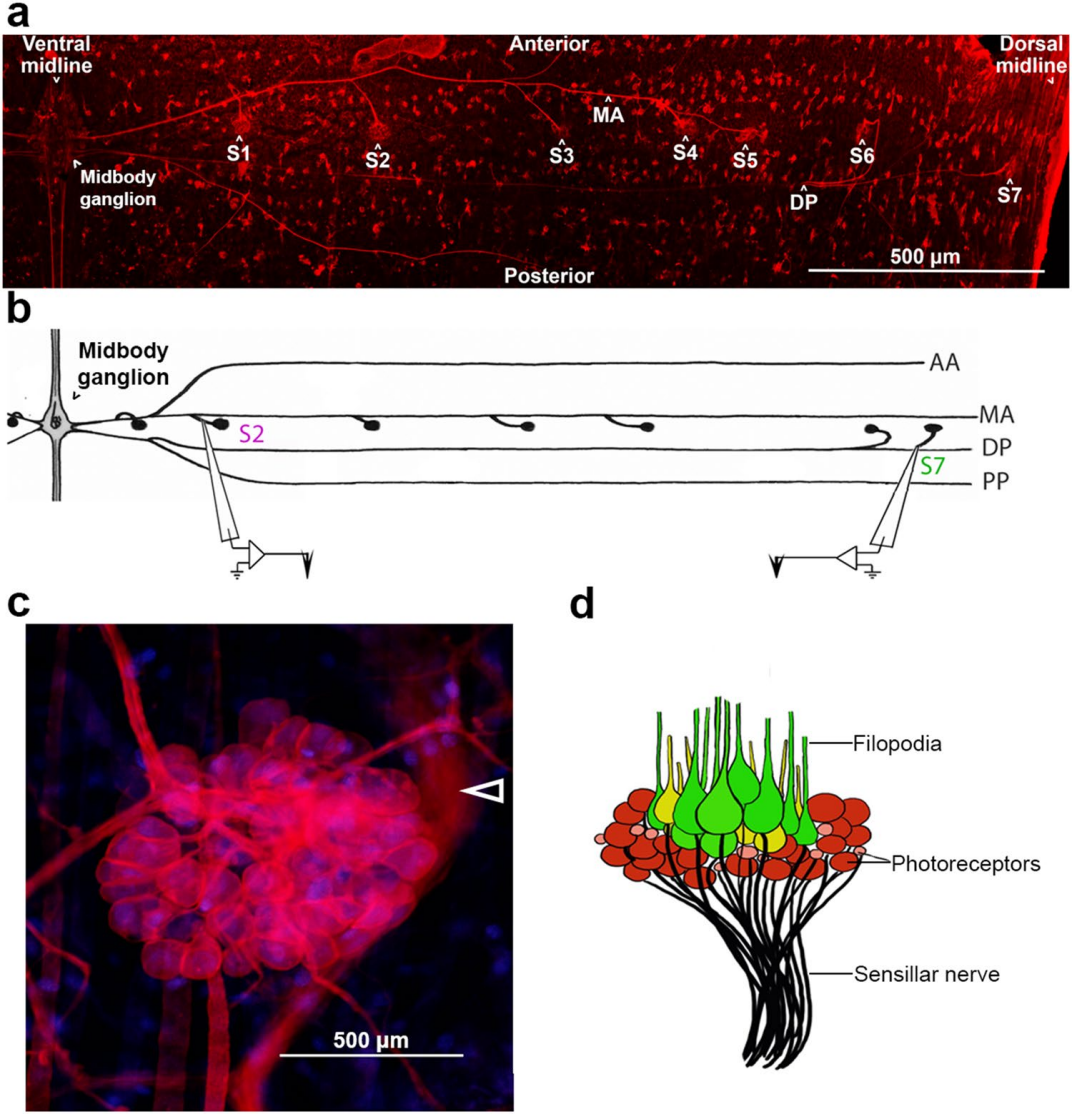
Leeches have 7 bilaterally paired dermal sensilla along the central annulus of each mid-body segment. **a** Confocal imaging of the right side of a mid-body segment with all 7 sensilla (S1–S7) labeled with Lan3-2 along with the medial (MA) and dorsal nerves (DP, partially out of focal plane in this image) that carry their axons to the segmental ganglion. **b** Hand drawn map of Fig. 2a depicting subsidiary nerves emanating from the anterior and posterior nerve roots of a segmental ganglion (along with labels for the anterior bifurcation [AA] of the anterior root and the posterior bifurcation [PP] of the posterior root). Individual sensillar nerves from ventral (S2) and dorsal (S7) sensilla are severed from the subsidiary branches of segmental ganglia and suction electrodes are used for extracellular recording from the severed sensillar nerves. Individual sensilla are depicted by black dots. **c** Confocal imaging of the S1 stained with DiI (red) and bisBenzimide (blue) and **d** illustration of the same sensillum, showing the arrangement of cells and the sensillar nerve head used for collecting population responses

The medicinal leech extracts visual information from nearly every angle of the 3-dimensional space surrounding the animal using two discrete populations of eyespots. One set of visual inputs consists of 5 pairs of cephalic pigmented eyecups, each containing roughly 50 photoreceptors (Kretz et al. 1976). This array of cephalic eyes is oriented such that different eyes sample either the forward or dorsal visual field. Photoreceptors in the cephalic eyes (and dermal sensilla) are phaosomal primary receptor neurons that depolarize in response to light, producing action potentials that are conveyed by axons of the primary receptors into the central nervous system (Lasansky and Fuortes 1969; Fioravanti and Fuortes 1972; Kretz et al. 1976; Peterson 1984). Another set of visual inputs, the focus of the present work, consists of a widely distributed array of 7 bilateral pairs of dermal sensilla in each mid-body segment (Fig. 1), positioned dorsal to ventral along the central annulus of each mid-body segment (Kretz et al. 1976). In addition to photoreceptors, the dermal sensilla contain a number of ciliated mechanosensory cells (Derosa and Friesen 1981). Individual dermal sensilla are thought to contain fewer photoreceptors than cephalic eyes and project axons to the segmental ganglia via subsidiary branches of the anterior and posterior nerve roots (Kretz et al. 1976) (Fig. 2). The dermal sensilla have wide acceptance angles of ~ 100° and are oriented such that some of them survey dorsal, lateral, or ventral visual fields—meaning that they can possibly provide low-resolution directional selectivity for visual cues across the dorsal–ventral axis, originally suggested by Kretz et al. (1976).

Leech photoreceptors were known to respond maximally to green light (Kretz et al. 1976); however, Jellies (2014a) revealed a second peak of robust visual response in the ultraviolet (UV, 365–375 nm) range. The leech has been shown to avoid dorsally presented UV illumination by shortening away from UV presented at the head and extending away from UV presented at the tail while not showing a withdrawal from comparable levels of green or white light (Jellies 2014a). These integrated responses suggest that the leech can distinguish green and UV and direct different responses depending upon where the visual information arises on the body surface. Given that hirudiniid leeches are positively phototactic when hungry and use photic cues to find prey (Dickinson and Lent 1984; Harley et al. 2011), while simultaneously avoiding excessive amounts of light, (the tissue damaging ultraviolet) our working hypothesis is that the leech evolved discrete populations of photoreceptors that mediate either primarily UV or visible light detection. If these populations of photoreceptors were distributed differentially across the body axes, they might play a fundamental role in inputs that inform behavioral responses. In an extension of the initial studies, Jellies (2014b) showed that leeches would rotate away from UV light presented ventrally, but not dorsally. Further, leeches ignored green light presented ventrally in behavioral tests. Given that leeches lack specialized statocysts (Mann 1962), Jellies (2014b) suggested that differential responses to wavelength of light along body surfaces may be used as a spectral statocyst to guide a dorsal light reflex (DLR) of body position in 3-dimensional space. The primary purpose of the present studies was to examine the sensitivity of dermal sensilla to the wavelengths of light previously shown to evoke the orientation behavior.

When the leech’s prey moves through water it can generate surface waves. The curved surfaces of these waves act as converging lenses and produce pulses of downwelling irradiance which appear as moving bars of light. *Hirudo* uses vision to orient to these moving bars of light originating from potential prey (Dickinson and Lent 1984; Harley et al. 2011). Although mechanical cues generated by water waves are an effective stimulus for orientation, bars of light generated by these water waves are themselves effective for orientation even in the absence of mechanical cues from surface waves. In addition, quiescent leeches exhibit a stationary rhythmic undulation, or ventilation, and freeze in response to moving shadows or bars of light that might be correlated with a looming predator (Mann 1962). Based on our preliminary studies, the complex response of individual sensilla to light stimuli showed modest phasic and tonic components. The phasi-tonic nature of these receptors may enable them to encode both dynamic changes in stimuli (i.e., increases in light intensity) and duration or removal of stimuli (Barth 1964; Randall et al. 2002). A secondary purpose of the present studies was to examine dermal sensilla for spatial mapping of adaptation properties that might permit encoding changes in luminance and provide information about leading and trailing edges of moving bars of light selectively in the visual field.

## Methods

### Animals

Originally acquired from Leeches USA (Westbury, NY, USA) and Niagara Leeches (Cheyenne, WY, USA), medicinal leeches (*Hirudo* spp.) were used to establish an in-house breeding colony housed in aquarium tanks at room temperature (20 °C). Cocoons are opened after 30 days and juvenile animals are transferred to 60X15 mm Petri dishes containing artificial pond water [Instant Ocean sea salt (Spectrum Brands Inc., Madison, WI, USA) diluted with spring water to a concentration of 0.05%]. Unlike the breeding colony, the animal colony was housed in aquaria (filled with artificial pond water, Instant Ocean diluted 1:100 in purified water) and maintained at 15 °C. Adults fed 2–3 times and older than 1 year were used for electrophysiology and dye-filling. One unfed embryo was used for immunohistochemistry. A total of 30 adult animals were used in experiments to assess mapping of spectral sensitivity and adaptation properties in the dermal sensilla.

### LED stimuli

**Fig. 3:**
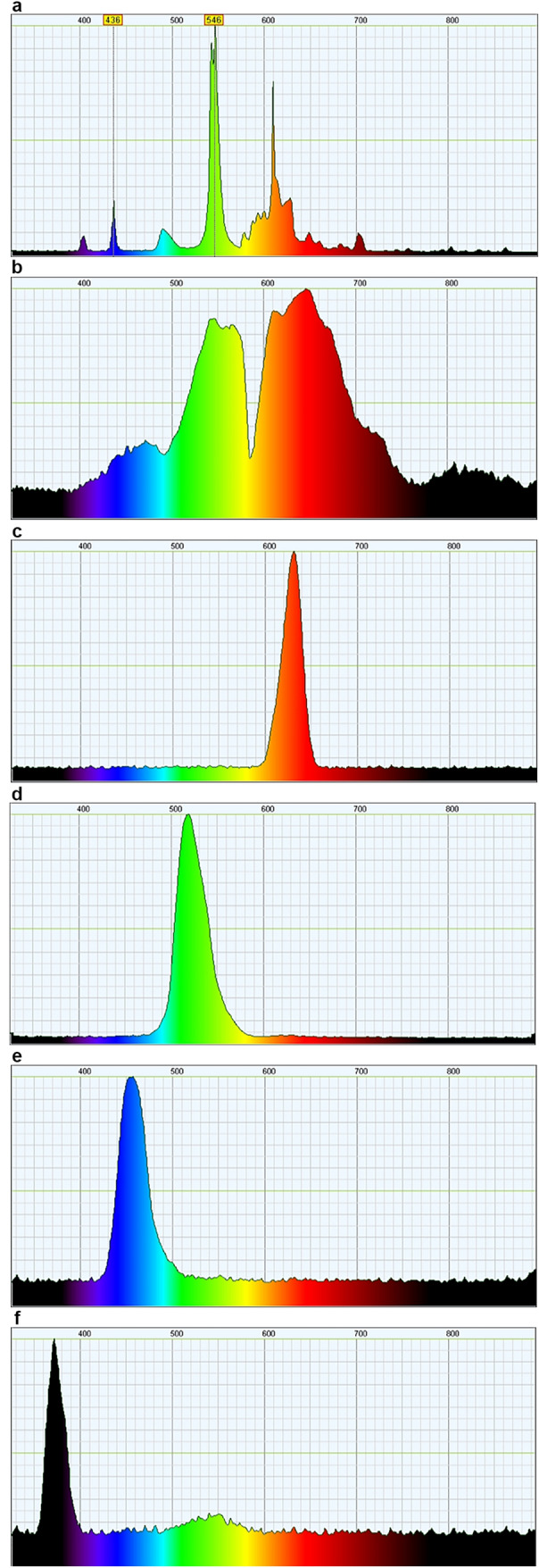
Spectrophotometer characterization of stimuli used in the present study. **a** The Theremino-based spectrophotometer used in this study was calibrated against the main mercury lines of a compact fluorescent bulb. Once calibrated, we employed the spectrophotometer to confirm the emission profile for each stimulus used, including **b** the 50 W halogen bulb and the red (**c**), green (**d**), blue (**e**), and UV (**f**) LEDs

To survey the mapping of spectral sensitivity, LEDs of four different nominal wavelength ranges were used as stimuli: red (615–640 nm), green (515–520 nm), blue (460–470 nm), and UV (365–375 nm). These LEDs were obtained and characterized in accordance with our previous report (Jellies 2014b). Each LED wand contained fixed resistors to limit current, and an RCA plug at the end of the wand for attachment to a 9 V power source provided by an ADI PowerLab 26 T (ADInstruments, Colorado Springs, CO). LED wands were engineered in a manner consistent with previous reports (Jellies 2014b), such that each delivered approximately equal quantal flux over narrow wavelength bands. In other words, each wavelength was similarly *intense*. The LED emissions were characterized using a Theremino-based handmade spectrophotometer (Theremino-System is licensed under Creative Commons License). The spectrophotometer employed a 1000 line/mm diffraction grating and a sensor made from a 2 MP Logitech camera from which the infrared filter had been removed. It was calibrated against the main mercury lines of a compact fluorescent bulb according to instructions from Theremino (Fig. 3a). The halogen fiber optic used to project white light in this study had the expected profile (Fig. 3b). As anticipated, the emission envelopes of the LEDs used in this study were confirmed to be directly comparable to those used in the earlier studies (Jellies 2014a, b) (Fig. 3c–f).

Responses across a range of optical densities (OD, plotted as |log % transmittance|) were examined using a set of four neutral density (ND) filters with nickel–chromium-coated fused silica capable of reducing the transmittance of UV and visible wavelengths equally. These ND filters were placed between the preparation and the stimulus by inserting them into the open end of a simple holder containing one of four calibrated LEDs. In some cases, ND filters were stacked together in the holder to achieve a greater OD. When necessary, a 50 W halogen bulb projected though a fiber optic served as a white light stimulus. Each stimulus presentation was made from the same position for each of the ND conditions.

### Extracellular recordings

Prior to dissection, all animals were anesthetized with ice-cold isotonic salt solution (containing 115 mM NaCl, 4 mM KCl, 1.8 mM CaCl2, 10 mM d-glucose, 4.6 mM Tris maleate, and 5.4 mM Tris base) at a pH of 7.4 (Nicholls and Baylor 1968). All electrophysiology experiments were performed in low lab ambient light (Jellies 2014a) but not under dark-adapted conditions. Suction electrodes were used to obtain population responses of primary receptors in dermal sensilla and were amplified with an A-M Systems model 1700 differential amplifier (A-M Systems, Sequim, WA) with the gain at 1000×, low end filter at 10 kHz, and high-end filter set to 10,000 Hz. Recordings were captured with an Apple Mac Mini coupled with an ADI PowerLab 26 T sampling at 10 kHz.

**Fig. 4:**
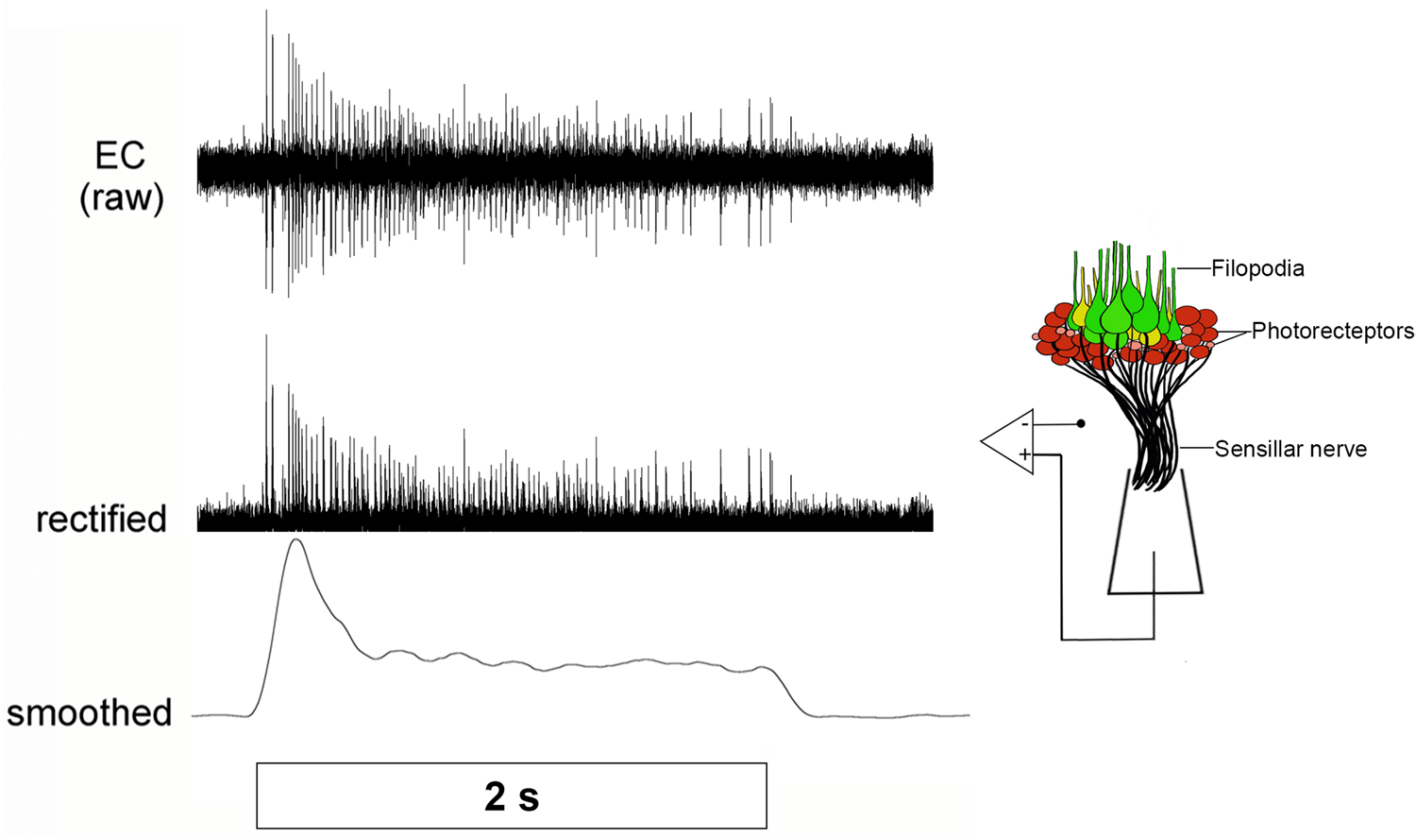
Raw extracellular recording (EC, top trace) of a representative sensillum (artistically depicted with suction electrode in the right panel) showing the complex spiking activity produced by the population of phaosomal photoreceptors in response to a 2-s pulse of white light. Population responses were rectified (middle trace) and smoothed (bottom trace) for each stimulus presented

To expose subsidiary identifiable branches of the segmental nerves that carry axons from ventral and dorsal sensilla, leeches were dissected with a dorsal midline incision and pinned dermis-down in a 60 mm dish filled with a 3 mm layer of clear Sylgard resin. These preparations isolated mid-body segments 6–11. Topographical mapping of the electrophysiological response characteristics of dermal sensilla was accomplished by monitoring complex population spiking responses from the photoreceptors in dorsal (S7) and ventral (S1, S2) sensilla (Fig. 4). These primary photoreceptors are of rhabdomeric origin that depolarize and produce action potentials in response to light (Kretz et al. 1976; Döring et al. 2013). To assess spectral sensitivity, we evaluated responses to a 2 s light pulse across wavelength and intensity in stimulation sequences of red, green, blue, and UV at different luminosities (0 OD, 0.3 OD, 1.3 OD, and 2.0 OD) using ND filters to regulate the amount of light.

Unlike other extracellular (EC) nerve recordings in the leech, Kretz et al. (1976) recognized that EC recordings from optic and sensillar nerves represented the summed activity of a relatively homogenous collection of several dozen axons firing action potentials coincident in time. Therefore, it was not possible to resolve individual spikes reliably that could otherwise be used to assess the response frequency of individual sensillar photoreceptors and a method was developed to quantify the aggregate response of these populations of primary receptors. The amplitude and duration of the recorded voltage excursions are not “spikes” in that they do not reflect single unit recordings, but rather the amplitude and duration of these events are directly related to the frequency and timing of coincident action potentials that underly each excursion in the record. As in Kretz et al. (1976), we quantified responses by determining the gross electrical charge passing into and out of the sensillar nerve during the stimulus presentation. First, we rectified each response by taking the absolute value of the voltages in the raw EC record (Fig. 4). The rectified data were then smoothed using the Triangular (Bartlett) window smoothing function in LabChart (ADInstruments; Fig. 4). Lastly, we took the integral under the smoothed curve for each stimulus presentation and subsequently compared these values across spectrum, intensity, and location, as had been done previously in this system (Kretz et al. 1976). Integrating the complex spike trains in this way allowed for direct comparisons between conditions since the integrated signals are directly proportional to both frequency and duration of responses. Since integration combines frequency and duration, these data are not optimal to examine details of sensory adaptation. Nonetheless, we were able to make a simple overt comparison of response properties as crude estimates of temporal sensory adaptation. During these preparations, we used a 2-s pulse of white light to assess adaptation properties of the sensilla. Adaptation was quantified as a percentage of the peak response following stimulus presentation. To do this we measured ratio of the integral 1-s post-stimulus to the integral of the peak response.

### Dye-filling

To reveal the anatomy of an individual sensillum (as in Fig. 2c), one animal was dissected in cold isotonic salt solution and pinned dermis side down in a 60 mm dish filled with a 3 mm layer of Sylgard resin. With endothelial gut lining removed, the animal was fixed overnight at 4 °C in 4% paraformaldehyde (pH 7.4) and then rinsed in PBS. Visualization of individual sensillar cells was accomplished by pressure-injecting the lipid soluble dye, DiI (Molecular Probes), into a mid-body segmental ganglion. For this injection we used 10 mg of DiI in a solution containing 100 μL of DMSO and 50 μL of absolute ethanol. Nuclei were labeled by a further 24-h incubation in bisBenzimide (Sigma) stock at 10 mg/1 mL HPLC grade H_2_O which was diluted in PBS (1:1000). This preparation was then viewed with a Nikon Eclipse Ti confocal microscope using NIS Elements software (Nikon; Fig. 2c).

**Fig. 5:**
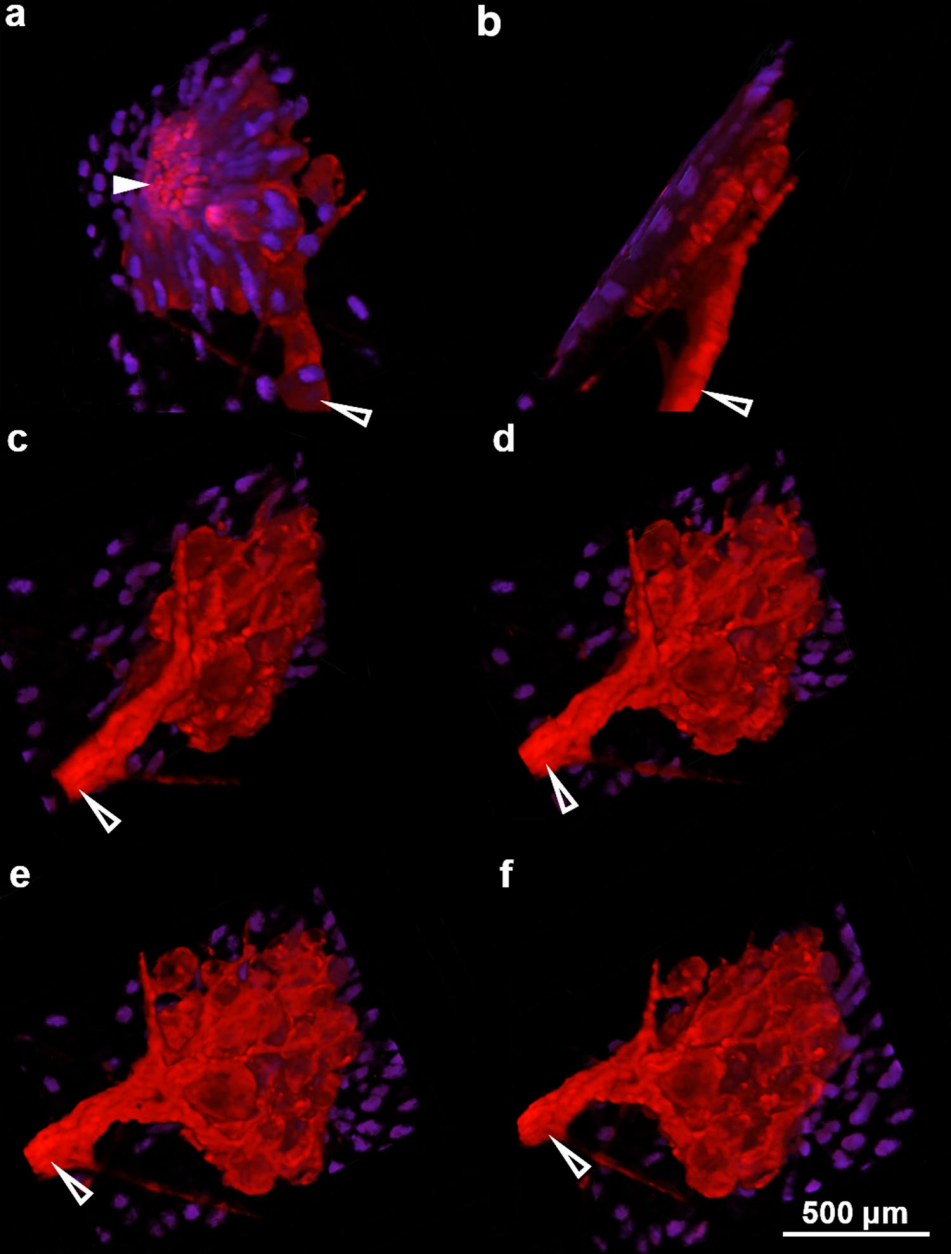
Alpha blending of a series of z slices captured by a confocal microscope produces a 3-dimensional sensillum with accentuated surface details. **a**–**f** Panels depict the rotation of a 3-dimentional sensillum, first viewing from the epithelial surface (**a**) and rotating to end with a view of the base of the sensillum (**f**). Open white arrows indicate the sensillar nerve, with the solid white arrow pointing to the cluster of filopodia. The filopodia in **a** are truncated due to limited sampling range along the z-axis. Note that the nuclei (planar organization of small blue dots) are only visible in overlying epithelial tissue, as the alpha rendering mode hides internal features of sensillar cells that are stained with DiI

We were able to provide further anatomical detail of a typical S1 sensillum by taking the same DiI preparations described above and using a Nikon Eclipse Ti confocal microscope to collect a series of images within the same *x*- and *y*-coordinates, but along the entire *z*-axis of the sensillum from superficial to deep using the *Z*-stack function in NIS Elements software (Nikon). Using this data, we assembled the *Z* slices into a 3-dimensional model that better-accentuated object surfaces by the applying alpha blending mode in NIS Elements software (Nikon; Fig. 5a–f).

## Immunohistochemistry

To visualize all segmental sensillar nerves (S1–S7, as in Fig. 2a) we labeled a leech embryo (embryonic day 16) with the Lan3-2 antibody (Zipser and McKay 1981; McKay et al. 1983). The monoclonal antibody Lan3-2 binds to a surface epitope of a membrane-associated antigen known to be expressed by all sensillar afferents (Johansen et al. 1992). The dissected embryo was fixed overnight at 4 °C in 4% paraformaldehyde and then incubated at room temperature for 48 h with diluted Lan3-2 antibody (1:75) in PBS with 1% Triton X-100, 10% normal goat serum, and 0.001% sodium azide. The preparation was then incubated in a Texas red-conjugated rabbit anti-mouse IgG_1_ secondary antibody (Molecular Probes) at a dilution of 1:250 for 48 h. The preparation was dehydrated in alcohol, cleared in methyl salicylate, and then embedded in DPX mountant between 2 pieces of cover glass. The preparation was then photographed (Fig. 2a) with a Nikon Eclipse Ti confocal microscope using NIS Elements software (Nikon).

### Data analysis

Analysis of spectral responses from dorsal and ventral sensilla was based on the recordings from 7 individuals (*n* = 7) for each spatially segregated input (14 animals in total) and analyzed using 2-way repeated measures ANOVA, with wavelength and optical density as the independent variables. All responses were computed by integrating the absolute values of the voltages recorded by the electrode during the 2-s stimulus presentation and normalized to the peak frequency within animals for each wavelength and optical density. Possible differences between means were determined by a post hoc test (Fisher’s LSD). Analysis of adaptation properties was accomplished by comparing attenuation from the peak response at 1-s post-stimulus presentation between ventral and dorsal sensilla. Analysis was based on the recordings from 8 animals (*n* = 8) from each subset of sensilla. Again, responses were computed as the integral of the absolute values of the voltages recorded by the electrode during white light presentation. Differences between the mean attenuation ratios were determined using an independent sample *t* test. For all tests, *P* < 0.05 is considered statistically significant.

## Results

### Anatomy of dermal sensilla

To visualize the anatomical location of the segmental nerves carrying afferent sensillar axons, we used the monoclonal antibody Lan3-2 (Zipser and McKay 1981; McKay et al. 1983) to label all sensillar afferents. In this case, Lan3-2 was bound to a surface epitope of a membrane-associated antigen that is known to be expressed by all sensillar afferents (Johansen et al. 1992). This technique allowed us to confirm the location of sensillar afferents in the subsidiary nerves branching from the anterior and posterior nerve roots of mid-body segmental ganglia (Fig. 2a). Using a simplified, hand-drawn map of the anatomy revealed by Lan3-2 (Fig. 2b), we were able to readily find, dissect, and prepare each subsidiary nerve for EC recording.

Adding to the morphological detail of dermal sensilla as presented by Derosa and Friesen (1981), we used a dye-staining technique to label the cell membrane of sensillar neurons (Fig. 2c). Using the lipid soluble dye, DiI, we revealed the circular arrangement of spherical sensillar neurons around a core of cells projecting long hair-like processes from the epithelial surface. This arrangement is further clarified by an artistic interpretation of the aggregate mid-body sensillum as revealed by this dye-filling process, presenting the putative location of sensillar photoreceptors (Fig. 2d). We also constructed a digital 3-dimensional representation of a typical ventral sensillum by stitching together a series of images taken along the superficial to deep axis (z-axis) of a representative S1 sensillum (Fig. 5a–f).

### Dorsal sensilla respond most strongly to green light

**Fig. 6:**
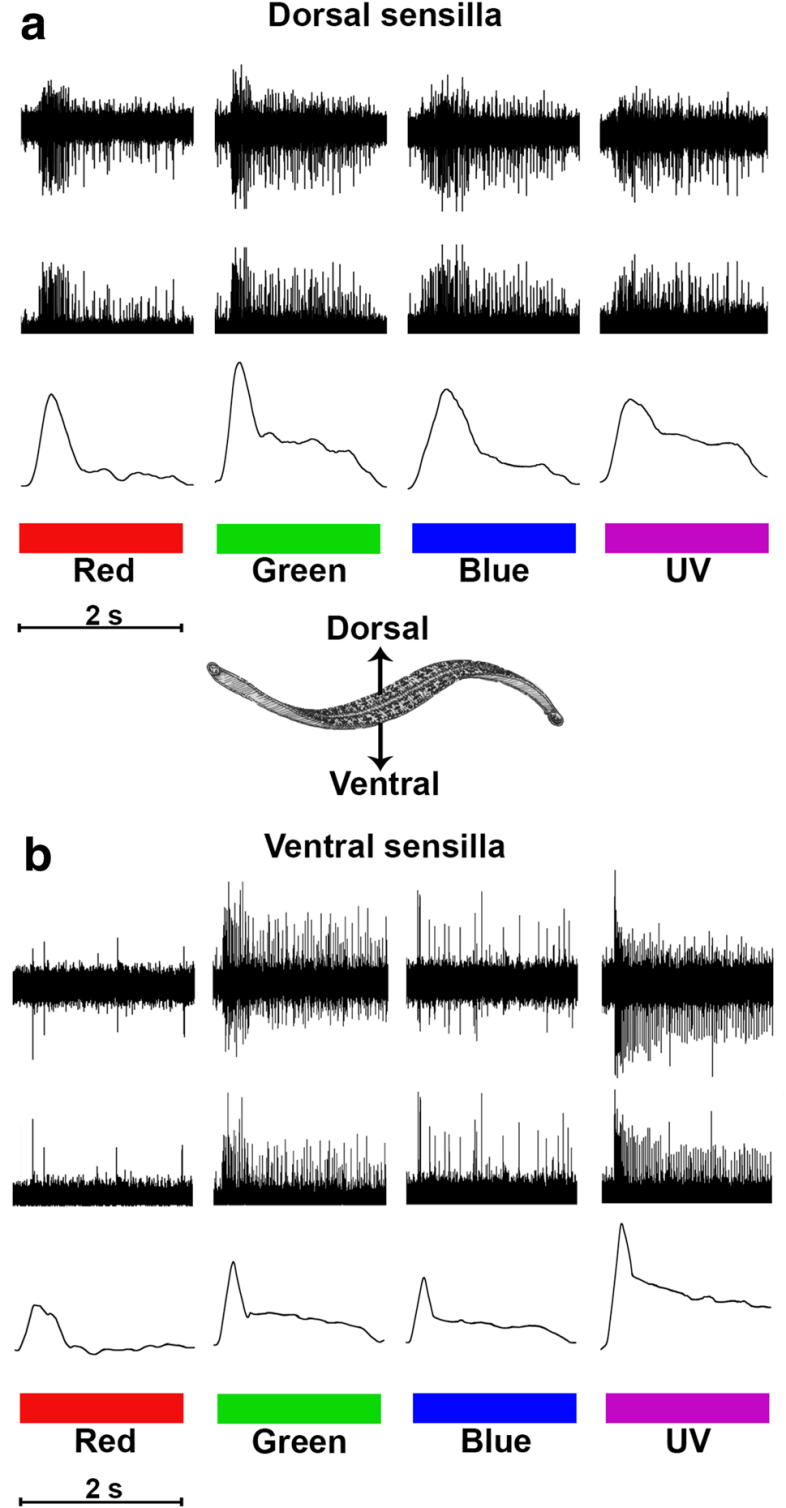
Extracellular recordings of sensillar nerves are used to evaluate responses to a 2-s light pulse across wavelength and optical density (OD; |log % transmittance|) in stimulation sequences of red, green, blue, and UV. For each response in (**a**) and (**b**), spike trains (top trace) are rectified (middle trace) and smoothed (bottom trace) for comparison. Dorsal sensilla (**a**) appear to optimally respond to green light, while ventral sensilla (**b**) optimally respond to UV light

**Fig. 7:**
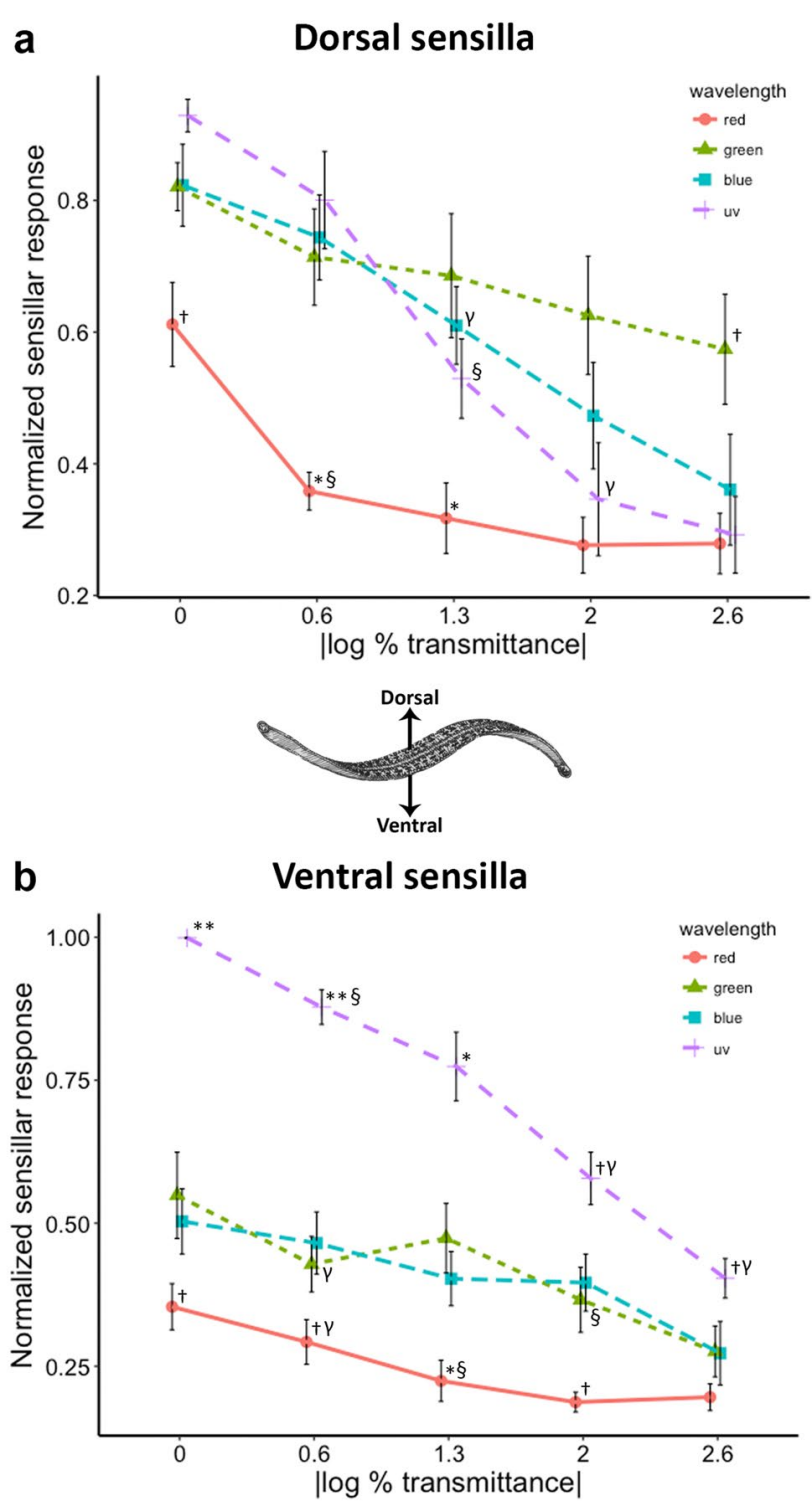
Integrating signals for each response and normalizing to the maximal response in each trial allows for direct comparisons between conditions since they are directly proportional to frequency and duration of responses. **a** Dorsal sensilla respond to green light and are most strongly activated by green light at OD 2.6. At OD 0, 0.6, and 1.3, responses to red are relatively small. **b** In ventral sensilla, UV light evokes the strongest response across all luminosities (|log % transmittance|), with red light evoking the weakest response. For both dorsal and ventral sensilla a two-way repeated measures ANOVA reveals significant main effects of both wavelength (*P* < 0.001) and luminosity (*P* < 0.001). Post hoc analysis shows significant differences at a given OD between wavelengths (**P < 0.001, *P < 0.01, †*P* < 0.05), and in some cases for a given wavelength between OD (§*P* < 0.01, γ*P* < 0.05)

A semi-intact preparation was used to expose sensillar nerves (Fig. 2a, b) in mid-body segments 9, 10, or 11 for EC recording of dorsal and ventral photoreceptors (Fig. 6a, b). Sensillum 7 (S7) was used in the dorsal preparations to assess visual input from the dorsal visual field, while sensilla 1 or 2 (S1, S2) were used in ventral preparations to similarly assess visual input from the ventral visual field. To determine the spectral sensitivity for ventral and dorsal sensilla, responses were compared between red, green, blue, and UV stimuli across a range of luminosity (0, 0.6, 1.3, 2.0, and 2.6 optical density). Analysis revealed that the dorsal sensilla are generally sensitive to visible light and exhibited a slightly stronger response to green at greater OD levels (Fig. 7a). While our data are not plotted as tuning curves (since we used discrete LEDs as stimuli), these responses are consistent with the dorsal sensilla being broadly tuned to visible light with maximal sensitivity near green wavelengths. For dorsal sensilla a two-way repeated measures ANOVA revealed a significant main effect of both wavelength (*P* < 0.001) and optical density (OD; *P* < 0.001). The two-way repeated measures ANOVA also revealed a significant interaction between wavelength and optical density (*P* < 0.05). Post hoc analysis (Fisher’s LSD) was used to assess differences between means, revealing that for 0, 0.6, and 1.3 OD there were no significant differences between the responses to green, blue, and UV, while red stimuli were least effective in generating a response (†*P* < 0.05, *P < 0.01). At lowest level of transmittance (2.6 OD), green was the most effective stimulus (†*P* < 0.05).

### Ventral sensilla respond most strongly to UV light

As in the dorsal sensillar recordings, a semi-intact preparation was performed to expose sensillar nerves from S1 or S2 (Fig. 2b), which represent input from the ventral visual field of the animal. Again, the spectral sensitivity for this population of dermal sensilla was determined by comparing responses to red, green, blue, and UV light across a range of luminosity. In contrast to the broad sensitivity of dorsal sensilla around green light, analysis revealed that ventral sensilla respond strongly and preferentially to UV light, with UV stimuli evoking far greater responses than other stimuli across all tested optical density levels, except for OD 1.3. A repeated measures ANOVA was performed and revealed main effects of both wavelength (*P* < 0.001) and optical density (*P* < 0.001). As was the case in the dorsal sensilla, a significant interaction between wavelength and optical density was revealed (*P* < 0.001). Post hoc analysis (Fisher’s LSD) was used to assess differences between means. At 0 and 0.6 OD, UV stimuli generated the largest response (*P* < 0.001), while red was the least effective stimulus at these luminosities (Fig. 7b; *P* < 0.05). At 2.0 and 2.6 OD, UV remained the most effective stimulus (*P* < 0.05). Green and blue stimuli were indistinguishable in their effectiveness across each level of OD. As expected, responses to a given wavelength exhibited a reduction from one OD level to the next (Fig. 7b; γ*P* < 0.05, §*P* < 0.01).

### Dorsally oriented sensilla did not adapt to white light faster than ventrally oriented sensilla

**Fig. 8:**
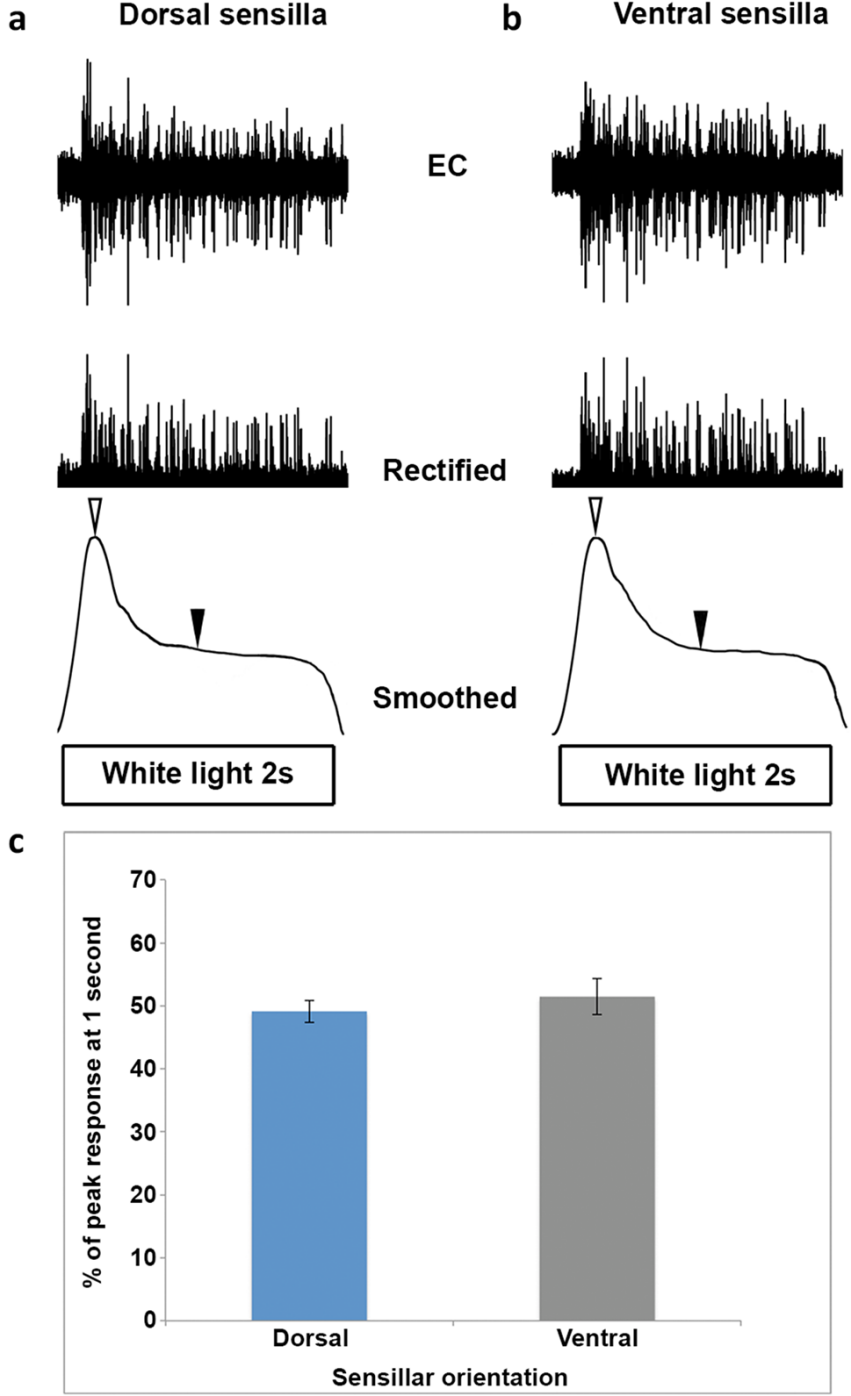
Dorsal and ventral sensilla all display a phasic-tonic response and are indistinguishable in terms of their overall adaptation properties. Responses to a two-second pulse of white light are rectified and smoothed (bottom trace) for comparison in both **a** dorsal and **b** ventral sensilla. **c** The average percent of the peak response (open arrows, bottom trace) at 1-s post-stimulus (closed arrows, bottom trace) is compared between groups. An independent samples t test does not reveal a significant difference between dorsal and ventral sensilla adaptation properties (*t* = − 0.69584, *P* = 0.4979)

Given that leeches respond to moving bars of light produced by the lensing activity of water waves originating from possible prey (Dickinson and Lent 1984; Harley et al. 2011), we hypothesized that the dorsally oriented sensilla might be specialized to extract this information from the dorsal visual field. To compare basic adaptation profiles of the sensilla, we employed the same preparation used to assess their spectral sensitivity and we measured the amount of reduction from the peak response to a white light stimulus following 1 s of stimulus exposure (Fig. 8a, b). Comparison of the mean attenuation between dorsal and ventral sensilla was accomplished with an independent samples t test. Comparison of the mean attenuation between dorsal and ventral sensilla to white light did not reveal a significant difference (Fig. 8c; *t* = − 0.69584, *P* = 0.4979).

## Discussion

According to Nilsson (2013), photoreceptor-controlled behavior can be broadly classified into 4 classes of behaviors, ranging from simple to progressively more complex. The simplest of these are the class I behaviors which represent nondirectional photoreception, such as entrainment of a circadian clock or simple shadow detection. Beyond class I are the behaviors influenced by directional photoreception (class II), such as postural orientation in relation to light (Nilsson 2009), followed by low-resolution vision (class III). Affording more than simple photoreception, low-resolution vision includes spatial resolution which can be provided by arrays of directional photoreceptors such as those found in compound eyes and even pigmented eyecups. This type of arrangement provides directionality by restricting the interaction of light with discrete regions of a photosensitive surface. Lastly, the most advanced photoreceptor-controlled behaviors are classified as high-resolution vision (class IV) and represented by animals capable of high spatial resolution. In one view, simple, non-image-forming eyes are expected to provide limited value scarcely beyond simple luminance detection (Land and Nilsson 2012). Refining this simplification, *Drosophila melanogaster* larvae were shown to use scanning by non-image-forming inputs to distinguish more complex image features (Justice et al. 2012). For an animal to be described as using low-resolution vision, Nilsson (2013) argues that animals equipped with directional photoreceptors must respond in a way that is directionally selective, for example, beyond a simple withdrawal reflex.

A dispersed visual system containing many non-image-forming units may collectively act as a large compound eye if the arrangement of the individual components allows the animal to surveil different regions its visual field. Blevins and Johnsen (2004) similarly described a dispersed visual system in sea urchins, where it provides spatial resolution and enables the animals to seek shelter. Using the medicinal leech (*Hirudo* spp.) as a model, we further elaborate the idea that dispersed, non-image-forming eyes may contribute to simple visual feature extraction beyond encoding light levels. We propose as a working hypothesis that distributed arrays of photoreceptors might serve as low-resolution inputs allowing simple image feature extraction by the central nervous system used to assist in orientation. The phylogenetic position of leeches is among a clade including oligochaetes and the Sedentaria (fan and tube worms) and closely related to the group Errantia (Polychaetes) (Kuo and Lai, 2018). Many members of the more ancestral group in Annelida display complex eyes, all 3 types of photoreceptors and complex visually guided behaviors (Purschke et al. 2006), that presumably have underlying complexity in neural circuitry associated with them. Therefore, in a comparative view, while the visual systems of various leeches may be more rudimentary than those of the ancestral groups, we hypothesize that at least some of the underlying neural circuitry subserving more complex visual analyses may have been retained or repurposed. This remains to be examined in further detail.

The results of these present studies show that the medicinal leech exhibits distinct topographical segregation of spectral sensitivity (sensory tuning) in the dermal sensilla across its dorsal–ventral body axis (Fig. 7a, b). The pronounced spatial segregation of these inputs, combined with previously reported differential activation of a higher order interneuron (Jellies 2014b), supports the hypothesis that spatially segregated spectral response, rather than luminance (brightness), may underlie reflexive dorsal–ventral body orientation in the leech. Further, it appears that this initial discrimination occurs at the level of primary sensory inputs. We also found that the ventral and dorsal sensilla did not diverge in their temporal responses to pulses of white light (Fig. 8c). So, while all sensillar visual responses contain both phasic and tonic components that might allow for detection of temporal variations, the dorsally oriented dermal sensilla may be no better (or worse) able to encode temporally repetitive cues to white light than the ventral sensilla.

### Leeches show topographical mapping of spectral responses across the dorsal–ventral body axis

Ultraviolet photoreceptors can be found in many taxa, extending the spectral range of color vision, which is commonly viewed as a strategy to provide additional contrast of visual scenes that contain UV light (Cronin et al. 2014). When presented with UV light from the ventral visual field, leeches specifically rotate away from it to place their dorsal midline directly toward the origin of the UV stimulus (Jellies 2014b). Yet, when UV was presented dorsally leeches maintain their dorsal aspect upward but move away from it by either contracting or extending, swimming or crawling but never rotating (Jellies 2014a). This suggests that rather than positioning the dorsal surface in line with intense light, leeches are positioning to avoid illumination by UV on the ventral surface. Many animals exhibit a dorsal light reflex (DLR) in which the body turns to position the dorsal surface upward (Preuss and Budelmann 1995; Deliagina et al. 2006; Glantz and Schroeter 2007; Schuster et al. 2011). In most cases this is driven toward the highest point of luminosity in the environment (von Holst 1950). However, for leeches this had never been shown, and until recently, luminosity was merely presumed to be the salient visual cue available to leeches, with maximal sensitivity to green light (Kretz 1976). However, the leech must be able to respond both positively and negatively to light depending upon context, complicating the idea that simple increases or decreases in brightness could convey unambiguous information. As shown in this present study, differential responses to UV and green light across the dorsal–ventral body axis (Fig. 7a, b) suggests that spatially segregated spectral responses, rather than luminance may be the major set of cues informing body position in *Hirudo*. *Hirudo* can hunt in daylight and as previously noted, employ visual cues in a positive fashion (being attracted to visible light), Mann (1962) noted that hungry medicinal leeches are positively phototactic. Yet, in the water and on land the leeches may need to maintain their counter shaded dorsal surface upward for camouflage. Consistent with our results, we speculate that leeches avoid presenting their more visually obvious and lightly pigmented ventral surface upward using the UV component of ambient light to inform the leech of the exposure of its ventral surface and trigger a positional adjustment.

Previous studies of the leech visual system showed that interneuronal targets differ for specific eyes (Peterson 1984, 1985). We predicted that cascading visual information into the CNS that causes rotation away from ventrally presented UV, but not green light, is the result primarily of spectral discrimination at the level of primary receptors in the sensilla, rather than interactions between central synapses. In a previous publication, a multimodal interneuron, the S cell, was shown to respond maximally to UV light when ventral sensillar pathways remained intact, yet it responded maximally to green light in conditions where the dorsal sensilla were intact (Jellies 2014b). Differential encoding of light signals between two widely spaced body areas, as presented here, imply that light-evoked rotational behavior is chosen primarily at the sensory level, rather than an interneuronal level.

Although determining the mechanisms responsible for phototransduction in this system is beyond the scope of this study, differential encoding of light on dorsal and ventral surfaces may be the result of variable expression of multiple opsins in photoreceptors such as those found in another distantly related leech species, *Hirudo robusta* (Döring et al. 2013). This is further supported by transcriptomic data revealing the presence of two putative opsins expressed in the body wall of *Hirudo verbana* (Stowasser et al. 2019)*,* where their comparison to opsins in other invertebrate species suggests that these are responsible for the distinct encoding of UV and green light across the body wall*.* Regardless of mechanism, our data show that all sensilla respond to green, blue, UV, and white light stimuli (Fig. 6a, b); however, the notable tonic nature of the response to UV light in ventral sensilla suggests that these sensilla may contain a population of photoreceptors that are uniquely responsive to UV (Fig. 7a, b), in that they (and not dorsal sensilla) show a sustained response that lasts well beyond the presentation of UV light (Fig. 6b). More extensive intracellular inspection of sensillar photoreceptors is necessary to determine the mechanism of spectral discrimination.

### Temporal response to white light does not differ between dorsal and ventral sensilla, but ventral sensilla show slow adaptation to UV light

The leech can integrate multimodal stimuli to locate prey, but this integration appears to differ between locomotor and non-locomotor modes of prey localization, such that the animal does not readily adjust to mechanical or visual stimuli during swimming (Harley et al. 2011, 2013). Indeed, the leech orients toward prey by directing its head toward the origin of prey-associated stimuli. Considering the lack of sensitivity of dorsal and ventral sensillar photoreceptors and the presumed dominance of cephalic eyes and sensillar mechanoreceptors in prey localization, we suspect that photoreceptors in the dorsal sensilla are not as useful in extracting repetitive stimuli.

While we found no clear difference between the ventral and dorsal sensilla in terms of their ability to adapt to white light (Fig. 8c), we did observe a prolonged response to UV light in the ventral sensilla. Recordings from ventral sensilla show a slowly adapting response to UV, with activity in the sensillar nerves far outlasting the stimulus presentation (Fig. 6b). Combined with previous reports of a tonic response of interneurons to ventral UV light in light adapted conditions (Jellies 2014b), we suggest that the ventral sensilla contain discrete photoreceptors that are more responsive to UV light than other populations of photoreceptors in the cephalic eyes and dorsal sensilla. Similarly, the median ocellus of horseshoe crabs (*Limulus*) is known to possess a discrete population of UV sensitive photoreceptors that exhibit a prolonged discharge continuing well beyond presentation of bright UV stimuli (Nolte and Brown 1972). Not unlike these UV sensitive photoreceptors described in *Limulus* (Nolte and Brown 1972), the prolonged discharge observed in *Hirudo’s* ventral sensillar nerves following UV exposure can be extinguished by a brief pulse of white light (not shown), suggesting an interaction between responses to UV and white light and the possible existence of a rhodopsin with photoactivatable intermediate states. This remains to be investigated.

Leeches are well suited to study the neural substrates responsible for coordinated movements (Kristan et al. 2005), and future studies must be conducted to determine how specific higher order interneurons, premotor interneurons, and motor neurons responsible for the rotation of the body are driven by the integration of UV and green light cues across the visual field. We have performed preliminary intracellular recordings of local premotor interneurons in the segmental ganglia to assess the possibility that symmetrical local circuitry is the neural substrate for asymmetrical rotational behavior. This small survey has yielded little evidence that local premotor interneurons switch their response patterns based on both stimulus wavelength and origin in the visual field, suggesting the possibility that ascending or descending inputs from distant interneurons impose local asymmetries that drive rotational behavior. Command neurons such as R3b-1, located in the cephalic brain of *Hirudo*, are involved in behavioral decisions (Puhl et al. 2012), and their sensitivity to light could have implications in local circuitry that drives coordinated rotation away from ventral UV stimuli. Further work must be performed to assess the contribution of such command neurons in the coordination of light guided behavior in *Hirudo*.

## Data Availability

Contact corresponding author for any data inquiries.
